# Diagnosis and Treatment of Acute Pancreatitis

**Published:** 1912-07-20

**Authors:** 


### DIAGNOSIS AND TREATMENT OF ACUTE PANCREATITIS.

The diseases of the pancreas are, at the present time, being subjected to an ever-increasing amount of clinical observation and to experimental research of the most varied forms. Especially is this true of acute pancreatitis, a disease which in its several varieties touches upon some of the most important problems with which the abdominal surgeon and the physiologist of to-day must deal. Acute pancreatitis is a rare disease, although close study reveals the fact that many cases must in former times have been overlooked. It presents difficulties in diagnosis and treatment, and it is but rarely that either the surgeon or the physician has the opportunity of seeing a large series of cases. The symptoms are those of an acute abdominal condition. Acute pancreatitis is to be suspected when a previously healthy person, or sufferer from occasional attacks of indigestion, is suddenly seized with violent pain in the epigastrium, followed by vomiting and collapse, and, in the course of twenty-four hours, by a circumscribed epigastric swelling, tympanitic or resistant, with slight rise of temperature.

The pain, as a rule, is severe, and generally referred to the epigastric region. It has been asserted by some that it is more often to the left of the median line, and while this may be a point of some value when present, the absence of localisation of the pain to the left side should not influence one in the least in estimating the importance of this symptom.

There is never any doubt concerning the severity of the pain wherever located. It practically always calls for the guarded use of opiates, although these are often without avail. Associated with the pain, but a far less constant symptom, is epigastric tenderness. In many cases it is but slightly marked, although careful examination will usually elicit it. It becomes more marked if the case progresses to suppuration, especially with the formation of a mass, which is practically always tender. In the same way epigastric muscular spasticity may occur as the result of the local condition.

Soon after the onset of the pain there is vomiting —a particularly constant symptom. The vomiting in some instances lasts but a few hours, or perhaps a day; and in others it becomes almost uncontrollable and progressively worse. This is a diagnostic sign of but little value, as it is common to most acute intra-abdominal conditions. Attention has also been called to the fact that it is, as a rule, biliary in character. Indeed, vomiting, especially when persistent, is one of the most misleading symptoms of the disease under consideration, as it often serves to make a diagnosis of intestinal obstruction seem the correct one. Fæcal vomiting, which is always present in the late stages of intestinal obstruction, is much rarer in acute pancreatitis.

### The Frequency of Collapse.

Persistent and uncontrollable belching and hiccoughing are marked symptoms of acute pancreatitis. They are often sufficiently severe to draw the physician's attention to the possibility of some trouble near the diaphragm, and to help to a correct diagnosis.

Collapse in the acute onset of pancreatitis is often very marked. In some of the cases, operated and unoperated upon, it may pass very quickly on to a fatal termination. In others, the patient recovers from it sufficiently to last for a few days, in very rapid cases, or even recovers therefrom entirely when a localised pancreatic suppuration supervenes. Fitz and others have called attention to the marked cyanosis which occasionally accompanies this collapse, and it has been referred to as a diagnostic feature. Its cause is still unexplained.

A possibly important symptom in the onset of acute pancreatitis is the frequent presence of a slow pulse even during the collapse, and until a septic condition supervenes. Indeed, it may even persist in these circumstances. If the patient survives the initial shock of hæmorrhages, as the case may be, certain other conditions make themselves manifest. There are evidences of intestinal paresis. Besides the vomiting and belching already spoken of, there is frequently, in the first two or three days, interference with the passage of gas and fæces. After several days repeated enemas will usually give quite copious and bulky stools.

### Presence of a Tumour.

Associated with these conditions there is intestinal and abdominal distension, with corresponding distress. Very often this seems to be largely colonic, affecting principally the transverse and descending colon, the small intestine being involved to a lesser extent. Slight jaundice is of frequent occurrence in pancreatitis. As a diagnostic sign it would serve only the purpose of calling one's attention to some lesion in the upper abdomen.

The formation of a more or less well-defined epigastric mass, apparently deep, usually somewhat tender, and often to the left of the median line, is very suggestive of pancreatitis, when found in conjunction with some of the other significant symptoms. Formerly, when cases were kept under observation longer because of failure to diagnose them and reluctance to operate except in the face of very definite indications, this was more often seen than it is now when the tendency is to operate somewhat earlier. Naturally the presence of a tumour such as this can hardly be expected in those instances of acute pancreatitis which quickly terminate fatally. The swollen, engorged, or hæmorrhagic pancreas itself is hardly ever palpable. The hæmorrhage in and about the pancreas scarcely ever gives such a considerable mass that palpation of it is possible; yet even this must be considered within the range of possibility. Finally, there is to be mentioned that occasional occurrence of dulness or of impaired resonance and breath sounds over the lower lobe of either lung, usually the left, may lead to a diagnosis of pleurisy as a complication.

The differential diagnosis is a matter of some difficulty in most cases. In the majority of instances in which an incorrect diagnosis has been made, it has been that of acute intestinal obstruction. It is not difficult to see why this is so. In both conditions there is the sudden onset, the violent, cramp-like pain, a slow pulse in the beginning, some distension, which gradually increases, and vomiting following the pain, with stoppage of gas and fæces. The similarity is heightened by the fact that in pancreatitis the distension is largely colonic, and this often leads to a suspicion of an obstruction low down.

### The Difficulty of Diagnosis.

At the onset of the disease it may be possible to differentiate the conditions, yet the uncertainty should not last very long. In the first place, it is rare to find shock, with occasional slow pulse and cyanosis in intestinal obstruction. The pain rather, where extremely severe, causes the patient to look blanched and exhausted. Moreover, it is, as a rule, localised to some point in the lower abdomen, rather than in the epigastrium, as in pancreatitis. Yet atypical locations thereof in pancreatitis are not entirely unknown. As the case progresses for some hours the distinction becomes slightly plainer. The vomiting in obstruction is one of the cardinal symptoms. It is persistent, severe, and becomes progressively worse. The vomit becomes foul in a short time. In pancreatitis the vomiting is bilious in practically every instance; after a short time it becomes less frequent. Far more distressing is the hiccoughing and belching associated with pain. They are not so prominent or striking in cases of obstruction. In pancreatitis the distension is generally colonic and not of high grade. In obstruction it may or may not be localised to the colon, is more marked, and more quickly progressive. In pancreatitis the ileus may be absolute at first, yet often high enemas result in the passage of gas and true bowel movements. In acute obstruction it is possible, by the same means, to obtain only emptying of the lower part of the larger bowel, and the passage of flatus, if it occurs at all, is very limited.

*(To be continued.)*

